# Role of Copper on Mitochondrial Function and Metabolism

**DOI:** 10.3389/fmolb.2021.711227

**Published:** 2021-08-24

**Authors:** Lina M. Ruiz, Allan Libedinsky, Alvaro A. Elorza

**Affiliations:** ^1^ Institute of Biomedical Sciences, Faculty of Health Sciences, Universidad Autónoma de Chile, Santiago, Chile; ^2^ Institute of Biomedical Sciences, Faculty of Medicine and Faculty of Life Sciences, Universidad Andres Bello, Santiago, Chile; ^3^ Millennium Institute on Immunology and Immunotherapy, Santiago, Chile

**Keywords:** mitochondria, copper, metabolic reprograming, hematopoietic stem cells (HSCs), cancer, ROS, proliferation, differentiation

## Abstract

Copper is essential for life processes like energy metabolism, reactive oxygen species detoxification, iron uptake, and signaling in eukaryotic organisms. Mitochondria gather copper for the assembly of cuproenzymes such as the respiratory complex IV, cytochrome c oxidase, and the antioxidant enzyme superoxide dismutase 1. In this regard, copper plays a role in mitochondrial function and signaling involving bioenergetics, dynamics, and mitophagy, which affect cell fate by means of metabolic reprogramming. In mammals, copper homeostasis is tightly regulated by the liver. However, cellular copper levels are tissue specific. Copper imbalances, either overload or deficiency, have been associated with many diseases, including anemia, neutropenia, and thrombocytopenia, as well as tumor development and cancer aggressivity. Consistently, new pharmacological developments have been addressed to reduce or exacerbate copper levels as potential cancer therapies. This review goes over the copper source, distribution, cellular uptake, and its role in mitochondrial function, metabolic reprograming, and cancer biology, linking copper metabolism with the field of regenerative medicine and cancer.

## Introduction

Mitochondria behave as biological microchips and constitute an integrated metabolic circuit that receives, processes, and transmits various signals to manage cell fate, that is, cellular proliferation, differentiation, and death. Beyond ATP and heat generation, mitochondria are directly involved in the epigenetics and transcriptional control of gene expression to rewire cell metabolism to new environmental conditions (Anderson et al., 2019; Hu et al., 2020).

The power of mitochondria and their ability to control cell metabolism and fate are based on the generation of the proton motive force (PMF) by the electron transport chain (ETC), which localizes in the inner mitochondrial membrane (IMM). The ETC is composed of the respiratory complex I (CI) NADH dehydrogenase, complex II (CII) succinate dehydrogenase, complex III (CIII) ubiquinol cytochrome c reductase, complex IV (CIV) cytochrome c oxidase, and the mobile electron carriers: coenzyme Q and cytochrome c. The ETC can be found in different configurations or arrays, varying from individual complexes to supramolecular associations, called supercomplexes or respirasomes, that are typically built of a monomer of CI, a dimer of CIII, and up to four monomers of CIV (I1III2IV0-4) (Schagger and Pfeiffer 2000; Boekema and Braun 2007; Huttemann et al., 2007; Acin-Perez et al., 2008; Dudkina et al., 2008). Unknown factors regulate the ratio between individual and assembled complexes, but it is likely to be dependent on cell physiology and energetic needs (Dudkina et al., 2008).

The proper assembly and functioning of the ETC is copper dependent (Kim et al., 2008; Turski and Thiele 2009). This transition metal is a prosthetic group of CIV and plays a direct role in the generation of the PMF. Furthermore, copper is a cofactor of the copper/zinc superoxide dismutase (SOD1), a protein located in both the cytosol and the mitochondrial inner membrane space to relieve the ETC-generated ROS (for the mitochondrial matrix ROS, there is an Mn-superoxide dismutase, SOD2) (McCord and Fridovich 1969; Yonashiro et al., 2009; Mondola et al., 2016). The indirect role of copper on mitochondrial function is related to the mitochondrial iron uptake since it is a cofactor of ferroxidases (Xu W. et al, 2013; Vallières et al., 2017). Iron transport is the key for iron–sulfur (FeS) cluster assembly and heme biosynthesis (Lill and Freibert 2020).

Hallmarks of dysfunctional copper metabolism are Wilson’s and Menkes diseases which are inherited disorders. Patients with Wilson’s disease accumulate copper in the liver due to the defective protein transporter, ATP7B. Copper overload produces liver cirrhosis, neurodegeneration, and anemia mostly *via* the exacerbation of hydroxyl radical production by the Fenton and Haber–Weiss reactions. Copper toxicity decreases mitochondrial respiration and induces apoptosis (Medeiros and Jennings 2002; Su et al., 2011; Jia et al., 2012). On the other hand, Menkes disease features mutations in the transporter ATP7A which affect the release of copper from the enterocyte to the bloodstream, causing a severe copper deficiency that in most of cases results in death due to CIV and SOD1 dysfunction (Rossi et al., 2004; Horn and Barrientos 2008; Kim et al., 2008; Turski and Thiele 2009). Common clinical manifestations of copper deficiency are anemia, bone marrow dysplasia, neutropenia, and neuromyelopathy (Gregg et al., 2002; Fong et al., 2007; Bolamperti et al., 2009; Jaiser and Winston 2010), and the appearance of enlarged mitochondria. These giant mitochondria or mega-mitochondria have been described in early precursors of the bone marrow, hepatocytes, and myocardium under copper deprivation, copper chelator treatments, and starvation in humans and rats (Dallman and Goodman 1970; Goodman et al., 1970; Gallagher et al., 1973; Wakabayashi 2002; Bustos et al., 2013; Ruiz et al., 2014). A mild copper deficiency in a murine model evoked mitochondrial adaptive responses involving the oxidative phosphorylation system (OXPHOS) remodeling and mitochondrial dynamic alterations, with upregulation of fusion proteins MFN-2 and OPA1 associated with a particular big mitochondrial morphology including normal and swollen mitochondria (Ruiz et al., 2014). Also, the generation of bigger mitochondria after copper deficiency in erythropoietic cells, cell line K562 and primary human CD34^+^, was related to the induction of mitochondrial fusion through upregulation of MFN-2 and OPA1 (Bustos et al., 2013).

Although heavy copper deficiency and overload have been associated with exacerbated ROS production and cell death due to mitochondrial dysfunction, noncytotoxic variations of the cellular copper levels may influence cell proliferation or differentiation through the reprogramming of mitochondrial metabolism, which can manage the balance between glycolysis and oxidative phosphorylation as well as the production of ROS, making the cellular environment oxidative (Bustos et al., 2013; Ruiz et al., 2016; Jensen et al., 2019). This exciting topic links copper metabolism with the field of regenerative medicine and cancer. In this review, we analyzed the acquisition of copper by the organisms and cells and the role of copper on mitochondrial function involving bioenergetics, dynamics, and mitophagy, and how this affects cell fate through metabolic reprogramming.

## Copper Uptake and Physiology

Copper is mainly obtained from solid foods (Supplementary Table S1, Supplementary Material) and drinking water (Council et al., 2000). The recommended intake of copper in humans should be less than 1.5 mg/d since 0.8 mg/d is enough to regulate and maintain copper status in the body. After the digestion of foods in the stomach and duodenum, copper is absorbed by cells of the intestinal mucosa, the enterocytes, which are also responsible for releasing copper into the blood plasma. When in excess, the ingested copper will be directly excreted through the feces, but when in lack, cellular mechanisms will be activated to allow greater intestinal uptake (Turnlund et al., 1998; Larin et al., 1999). The uptake efficiency of this metal is high, reaching 55–75% in adults (Johnson et al., 1992). Of note, copper concentration is tissue-dependent, varying between 3 mg (kidneys) and 46 mg (skeleton) (average adult ≈70 kg) (Linder 1991). Thus, the use of copper is different for each cell, and the effects produced by the imbalances of this metal will be tissue-specific. SupplementaryTable S1 (Supplementary Material) shows the amount of copper found in different kinds of food.

There are two phases for copper distribution into the organism (Owen 1971). Phase one: from food to the liver and kidneys *via* albumin and transcuprein. Phase two: from liver to other tissues and organs, such as heart, lungs, brain, and others *via* ceruloplasmin. The excess of copper must return to the liver, where it is again processed and incorporated into the bile which is the primary pathway for copper elimination from the body. However, some other fluids may transport copper to the intestine for excretion, such as the gastric and duodenal fluid. SupplementaryTable S2 (Supplementary Material) shows the concentrations of copper present in human fluids. Bile has the highest level of copper after blood. Copper entering the digestive tract through bile and other fluids becomes almost five times greater than copper consumed in the diet. However, only about 0.5–1.0 mg Cu is excreted. Most of it, about 4.5–5.5 mg Cu, is reabsorbed to maintain homeostasis of this metal (Linder and Roboz 1986).

**FIGURE 1:**
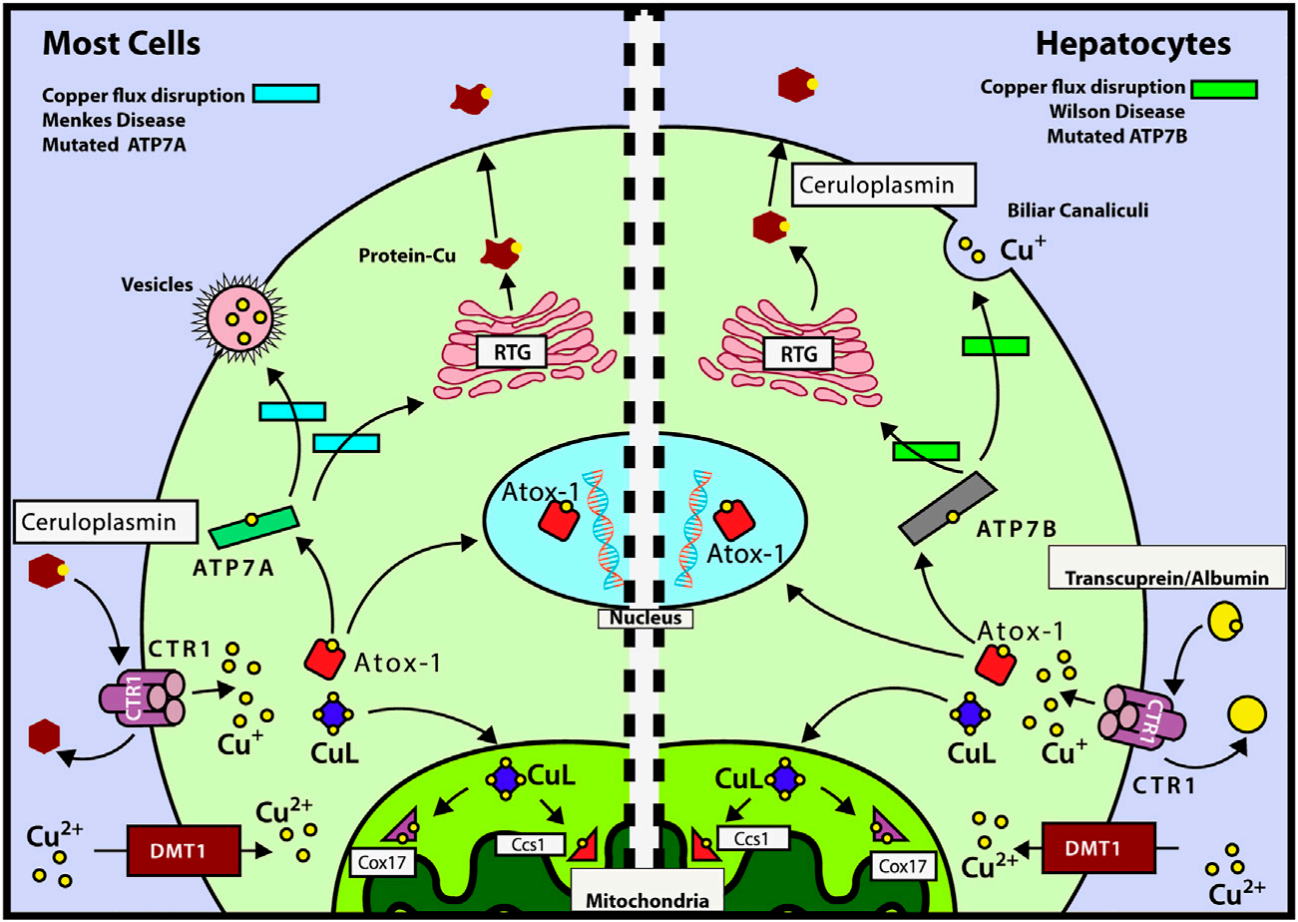
Mechanisms of intracellular copper transport. After Cu is delivered to CTR1 by transcuprein/albumin/ceruloplasmin, it is translocated to cytosol, where it can be transferred either to mitochondria by copper ligand CuL or incorporated into ATOX1. ATOX1 can translocate into nucleus, by a copper-mediated mechanism, and deliver its copper to ATP7A/B. **Left** panel: mechanisms of copper transport for most cells (except hepatocytes). ATOX1 delivers Cu to ATP7A which in turn transports it to the TGN or transfers it into vesicles to be excreted out of the cell. During copper deficiency and excess, metallothioneins MT-I and MT-II regulate ATP7A trafficking and control cell viability (Gudekar et al., 2020). **Right** panel: mechanisms of copper transport for hepatocytes. ATOX1 donates copper to ATP7B which transports it to the trans-Golgi network (TGN) for incorporation into ceruloplasmin. ATP7B is also responsible for the transport of Cu to the cellular periphery to be incorporated into the bile (main incharge of copper excretion). Also, we can observe the routes affected by the diseases of Wilson and Menkes in the green and light blue rectangles, respectively. Modified from the study by Fischer and Goode (1994), Puig and Thiele (2002), Cobine et al. (2006a), Cobine et al. (2006b), and Qin et al. (2008).

Copper is transported inside the cell by the CTR1 (copper transport protein) transporter and then delivered to ATOX1 (antioxidant 1 copper chaperone) and CuL (low molecular weight copper ligand). The latter transports copper into mitochondria, specifically to COX17 (copper chaperone of cytochrome C oxidase [COX]) and CCS1 (copper chaperone of SOD1) (Puig and Thiele 2002). Figure 1 shows the mechanisms of intracellular copper transport in human cells.

## Getting Copper Into Mitochondria

Mitochondria need copper mainly for complex IV (COX) and SOD1 among other proteins (Table 1). CIV has two copper ions and is involved in the generation of the PMF and the complete reduction of oxygen to water in the ETC; SOD1 has one copper ion and is an antioxidant enzyme dealing with anion superoxide. In eukaryotes, mitochondrial copper is stored in the cytosolic anionic ligand (CuL) complex, and its release requires high regulation for the efficient metalation of COX and SOD1. This highly regulated mechanism is probably due to the limited availability of this ion in the cytosol and the need for an immediate source of copper for the assembly of COX (Leary et al., 2009b).

**TABLE 1:** Copper binding and copper-dependent proteins key for the mitochondrial homeostasis.

Protein	Function	Subcellular localization	References
Cu/Zn SOD (SOD1)	Superoxide dismutase (SOD) catalyzes the reaction of superoxide to hydrogen peroxide and requires copper and zinc as cofactors	Mitochondria, cytosol and nucleus	McCord and Fridovich (1969); Yonashiro et al. (2009); Mondola et al. (2016)
Cytochrome c oxidase (COX)	The terminal enzyme in the mitochondrial respiratory chain catalyzes the reduction of dioxygen to water. Subunits 1–3 form the functional core of the enzyme complex. COXI is the catalytic subunit of the enzyme. Electrons originating in cytochrome c are transferred *via* copper a center of subunit 2 and heme a of subunit 1 to the bimetallic center formed by heme A3 and copper B	Inner Mitochondrial Membrane	Kadenbach and Hüttemann (2015)
COX17	Copper chaperone for COX. COX17 is a specific copper donor to Sco1 and Cox11, which donate copper to the CuB and CuA sites of cytochrome oxidase, respectively	Mitochondrial Intermembrane Space	Inesi (2016)
PIC2	A copper importer in the yeast mitochondrial matrix by the mitochondrial carrier family (MCF). Orthologues in humans SLC25A3 (copper/phosphate carrier protein, mitochondrial)	Inner Mitochondrial Membrane	Vest et al. (2013); Cobine et al. (2021)
MRS3	Yeast iron transporter, involved in mitochondrial copper homeostasis. Copper importer under copper-limiting conditions or in the absence of Pic2. Orthologues in humans SLC25A37 (mitoferrin-1) and SLC25A28 (mitoferrin-2)	Inner Mitochondrial Membrane	Vest et al. (2016)
SCO1	Metallochaperones whose principal function is to add two copper ions to the CuA site in the catalytic core of COX. There is a functional connection between SCO1 and CTR1: the high-affinity transporter that imports Cu into the cell. CTR1 degrades rapidly in the absence of SCO1 protein, suggesting a posttranslational mechanism to regulate CTR1-dependent Cu import into the cell through SCO1	Inner Mitochondrial Membrane	Hlynialuk Christopher et al. (2015); Morgada et al. (2015)
SCO2	Metallochaperones whose principal function is to add two copper ions to the CuA site in the catalytic core of COX. Copper-dependent thiol reductase of the cysteine ligands in the oxidase. Copper binding to Sco2 is essential to elicit its redox function and as a guardian of the reduced state of its cysteine residues in the oxidizing environment of the mitochondrial intermembrane space	Inner Mitochondrial Membrane	Morgada et al. (2015)
COA6	It is involved in the copper-dependent biogenesis of COX2. It can bind copper and can associate with newly translated COX2 and the mitochondrial copper chaperone SCO1	Mitochondrial intermembrane space	Stroud et al. (2015); Soma et al. (2019); Pacheu-Grau et al. (2020)
MIA40	Mitochondrial import and assembly protein, oxidoreductase of the mitochondrial disulfide relay, catalyzes disulfide bond formation in proteins in the IMS. By using copper-binding protein Cox17 as a natural substrate. Ccs1 shows interaction with Mia40. Mia40-mediated oxidative folding of domain I of Ccs1 may control the cellular distribution of Ccs1 and, consequently, active SOD1	Mitochondrial intermembrane space	Klöppel et al. (2011); Koch and Schmid (2014)
PARK7	Cu-dependent peptidase, function as a sensor of oxidative stress	Mitochondria, nucleus, endoplasmic reticulum and cytosol	Björkblom et al. (2013); Blockhuys et al. (2017)

Copper delivery into mitochondria is performed by the CuL, which is a non-proteinaceous low molecular weight complex found in the cytosol and the mitochondrial matrix (Leary et al., 2009b; Vest et al., 2013). The biochemical properties of the CuL are conserved from yeasts to humans (Cobine et al., 2004), debating that its functional significance started early in evolution (Leary et al., 2009b). It is currently thought that the binding of copper (copper-free) to the CuL in the cytosol triggers its translocation to the mitochondrial intermembrane space (IMS) by means of the solute carrier family 25 member 3 (SLC25A3), the mammalian homolog of yeast Pic2 (Leary et al., 2009b; Vest et al., 2013; Baker et al., 2017; Boulet et al., 2018; Cobine et al., 2021). CuL in the matrix is finally translocated across the IMM to IMS by an unknown transporter to metalate SOD1 and COX (Leary et al., 2009b; Baker et al., 2017).

In the mitochondrial matrix, copper is present in much higher quantities than required for metalation of COX and SOD1, suggesting that the matrix copper pool would represent a storage reserve for delivery to the IMS (Cobine et al., 2004). The ability of the CuL to increase or decrease its size due to changes in cellular copper levels (Cobine et al., 2004) indicates that the CuL may be a dynamic regulator that responds to changes in cellular copper concentrations (Leary et al., 2009b). This function allows mitochondria to assure the presence of a constant supply and reserve of copper for metalation of their biological targets, under different physiological processes (Leary et al., 2009b). The proper functioning of this signaling pathway also depends on proteins or secondary messengers that monitor and report the functional status of mitochondrial inner membrane proteins capable of sensing stimuli and transducing signals, such as copper-binding integral inner membrane proteins: COX11, SCO1, and SCO2 (Leary et al., 2009a; Leary et al., 2009b). Also, the COX assembly factors which are located in the IMS are soluble cysteine-rich proteins: COX17, COX19, COX23, and PET191 (Leary et al., 2009b). As a potential mechanism to stimulate OXPHOS metabolism, copper may directly act on the ETC to modulate the assembly or disassembly of respiratory complex IV. Indeed, complex IV serves as a metal sensor in the regulation of respiratory rates (Desler et al., 2012). Supporting this mechanism, copper deficiency reduces the expression and activity of complex IV (Dallman and Goodman 1970; Chen et al., 2002; Medeiros and Jennings 2002; Chen et al., 2005; Zeng et al., 2007; Ruiz et al., 2014), but not the other respiratory complexes (Zeng et al., 2007; Bustos et al., 2013). Regarding copper overload, the current results showed an increase in complex IV protein expression (Ruiz et al., 2016). Complex IV is made up of 11 protein subunits and requires 18 assembly factor proteins for accurate assembly (Koopman et al., 2012). A recent report showed that copper is able to rescue complex IV assembly in the mutant mouse deficient in the cytochrome assembly factor, COA6 (Ghosh et al., 2014). Therefore, copper helps to assemble complex IV to respond to energy demands. More complex IV assembly will enhance other complex assemblies, as has been reported for complex I (Diaz et al., 2006).

Copper deficiency, on the other hand, interferes with COX biosynthesis, producing a decrease in mitochondrial ATP production, which causes remodeling of ETC complexes, represses respiration, and creates a reduction in the efficiency of oxygen utilization (Kopp et al., 1983; Bode et al., 1992; Zeng et al., 2007; Shoubridge 2012; Bustos et al., 2013; Ruiz et al., 2014; Jensen et al., 2019). It has been observed that as COX biosynthesis increases, other complexes tend to increase, such as complex I (Diaz et al., 2006). In our laboratory, we observed that there is a considerable increase of all ETC complexes by adding a noncytotoxic copper concentration to K562 cells (Ruiz et al., 2016).

## Copper Has a Key Role in Cu/Zn SOD1 and COX Assembly

SOD enzymes have the primary responsibility for the dismutation of superoxide anions to hydrogen peroxide in cells. They have copper and zinc as a metal cofactor (SOD1 and SOD3), although there also exist variations with manganese in eukaryotes (SOD2) and with iron and nickel in bacteria and protists. In humans, there are three isoforms, that is, SOD1, SOD2, and SOD3. SOD1 is mostly located in the cytosol, but 5–10% can be found in the mitochondria. SOD2 localizes in the mitochondrial matrix and SOD3 in the extracellular fluid. Although SOD2 is the predominant superoxide dismutase in the mitochondrial matrix, SOD1 is critical to the control of oxidative stress in the mitochondrial intermembrane space (Zelko et al., 2002; Fukai and Ushio-Fukai 2011). The relevance of the antioxidant proteins and SODs is given by the control of ROS, which are known to regulate cell signaling (Wang et al., 2018).

**FIGURE 2:**
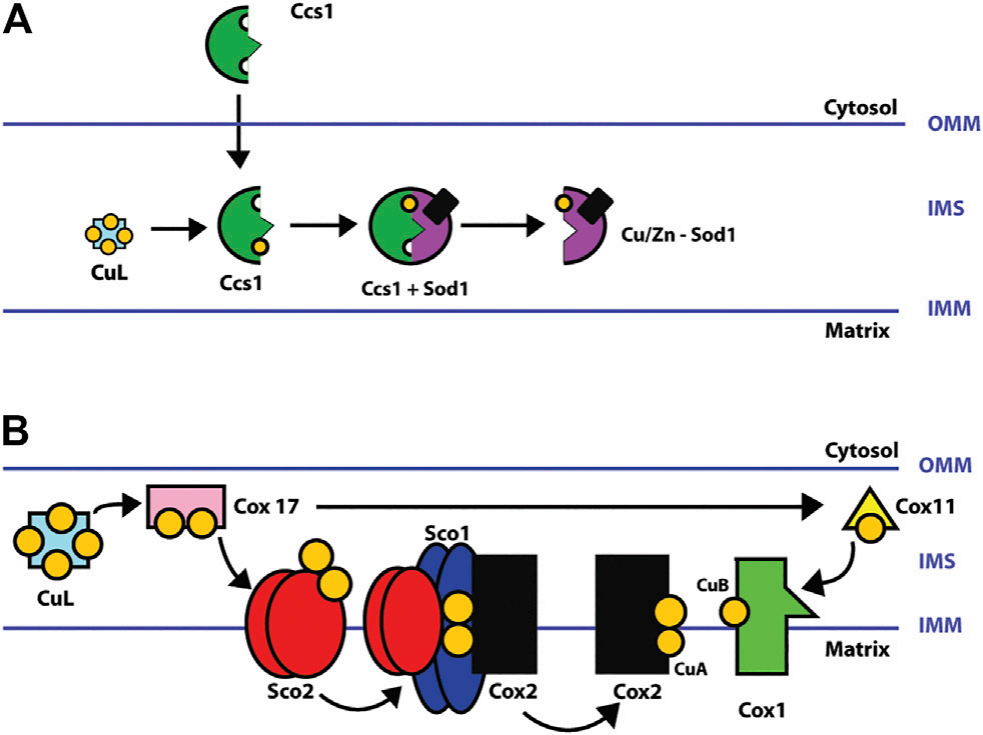
**(A)** Schematic of Cu/Zn-SOD1 metalation in CCS1 mitochondria. CuL transfers a copper ion to CCS1 in the intermembrane space. The incorporation of zinc may be during or before metalation with copper by CCS1. SOD1 is metalated in the intermembrane space (IMS) of mitochondria and then enters the matrix (Cobine et al., 2006a; Cobine et al., 2006b; Robinson and Winge 2010; Fukai and Ushio-Fukai 2011). **(B)** Metalation of COX copper centers (CuA and CuB sites). COX is assembled in parts that are then merged to produce the matured holoenzyme complex. The COX1 subunit contains the CuB site, and the COX2 subunit, the CuA site. After entering mitochondria, CuL donates two copper ions to the copper chaperone COX17 which in turn transfers the copper ions to COX11 and SCO2. COX11 metalates the CuB site of the subunit COX1, and SCO2, the CuA site of subunit COX2 by delivering two copper ions in a SCO1-mediated process (Robinson and Winge 2010; Jett and Leary 2018).

SOD1 is a homodimer having a binuclear copper and zinc site in each subunit, which forms a narrow channel where the dissociation of 2O_2_^−^ to H_2_O_2_ is carried out (Rhee et al., 2005). SOD1 is mostly metalated in the cytosol and remains in the cytosol. However, mitochondria-targeted SOD1 must first enter as an apoprotein (without copper) to be metalated in the IMS by CCS1 (Cobine et al., 2006a). CCS1 is a small polypeptide with three domains; domains 1 and 3 bind copper, while domain 2 is the key to the interaction with SOD1 (Lamb et al., 2000). The transient interaction is mediated by domains 2 and 3 of CCS1 (Robinson and Winge 2010). Figure 2 shows the place where CuL delivers copper to CCS1 inside the mitochondria.

COX is the terminal enzyme of the ETC and catalyzes the reduction of oxygen to water. It consists of 13 subunits. COX1 (MT_CO1), COX2 (MT-CO2), and COX3 are encoded in the mitochondrial DNA and form the COX catalytic center. The nuclear genome encodes the remaining ten subunits. The catalytic center of the enzyme contains 3 copper ions located in 2 copper centers, 2 copper ions in the CuA center, and 1 in the CuB center. Furthermore, two heme groups (α and α3) are also needed. The catalytic core is responsible for the oxidation of cytochrome c and the reduction of oxygen to water (Horn and Barrientos 2008; Leary et al., 2009b).

**FIGURE 3:**
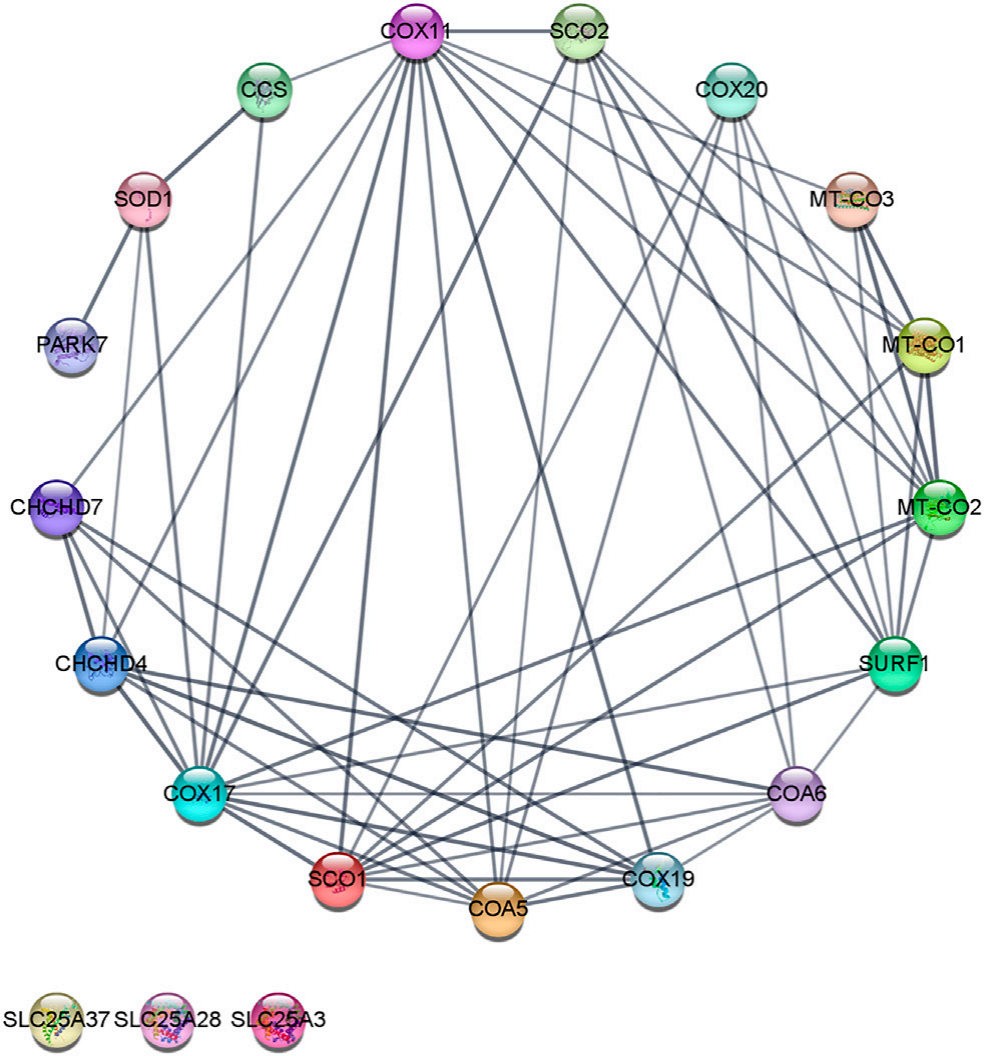
Copper protein network in mitochondria–STRING interaction network. The interaction network was created with the STRING (Search Tool for the Retrieval of Interacting Genes/Proteins) database version 11.0. A medium confidence cutoff of 0, 7 was implemented in this work. The resulting protein association network for copper was visualized by a Cytoscape v3.8.2. Proteins are presented as nodes (circles) connected by lines (Edge) whose thickness represents the strength of the connection based on the STRING database. For example, in the network’s inferior part, three nodes (“SLC25A28,” “SLC25A37,” and “SLC25A37”) are not connected to the network. The node “CCS” is connected to “COX11,” “COX17,” and “SOD1” nodes. The edge (line) that connected “CCS” with “SOD1” is thicker than the edge between “CCS” and “COX11,” and nodes (shared named, Stringdb canonical name, display name, neighborhood connectivity—number of neighbors). COX17 (9606. ENSP00000261070, Q14061, COX17, 7); COX11 (9606. ENSP00000299335, Q9Y6N1, COX11, 7); SURF1 (9606. ENSP00000361042, Q15526, SURF1, 8); PET191 (9606. ENSP00000330730, Q86WW8, COA5, 8); SCO1 (9606. ENSP00000255390, O75880, SCO1, 8); MT-CO2 (9606. ENSP00000354876, P00403, MT-CO2, 8); COA6 (9606. ENSP00000355572, Q5JTJ3, COA6, 8); MIA40 (9606. ENSP00000295767, Q8N4Q1, CHCHD4, 8); COX19 (9606. ENSP00000342015, Q49B96, COX19, 9); SCO2 (9606. ENSP00000444433, O43819, SCO2, 9); MT-CO1 (9606. ENSP00000354499, P00395, MT-CO1, 9); COX23 (9606. ENSP00000306425, Q9BUK0, CHCHD7, 8); COX20 (9606. ENSP00000406327, Q5RI15, COX20, 9); SOD1 (9606. ENSP00000270142, P00441, SOD1, 9); MT-CO3 (9606. ENSP00000354982, P00414, MT-CO3, 9); CCS (9606. ENSP00000436318, O14618, CCS, 6); PARK7 (9606. ENSP00000418770, Q99497, PARK7, 4); SLC25A3 (9606. ENSP00000228318, Q00325, SLC25A3, 0); SLC25A28 (9606. ENSP00000359526, Q96A46, SLC25A28, 0); and SLC25A37 (9606. ENSP00000429200, Q9NYZ2, SLC25A37, 0). In Supplementary Material additional data of the copper protein interaction network can be found.

COX17 initiates the copper transfer reactions for COX metalation in the IMS, which acquires copper from the CuL and requires additional COX11, SCO1, SCO2, and COA6. COX11 metalates the CuB site in COX1, and SCO1, SCO2, and COA6 metalate the CuA site in COX2. Once copper redox cofactors and heme groups are added into COX1 and COX2, the remaining subunits are added for final maturation (Figure 2) (Jett and Leary 2018; Robinson and Winge 2010). COA6 enables COX biogenesis as a thiol-reductase for copper to reduce disulfide bridges of critical cysteine residues in SCO1 and SCO2 metallochaperones in mitochondria (Pacheu-Grau et al., 2020; Soma et al., 2019). In this review, we analyzed the protein–protein interactions (PPIs) of the copper network in mitochondria with the Search Tool for the Retrieval of Interacting Genes/Proteins (STRING). The PPI network consisted of 20 nodes and 57 edges. The neighborhood connectivity of the nodes has an average of 7 neighbors (Figure 3). The interaction network shows that assembly and copper metalation of COX and SOD1 in mitochondria are very intricate. When COX1 is inserted in the mitochondrial membrane, SURF1 adds the heme group into COX1, and COX11 facilitates the formation of the CuB site. Copper for CuB is donated to COX11 by COX17 at the COX11 binding site right after cysteine sulfides are reduced by COX19. On the other hand, COX2 facilitates the maturation of the CuA site when it is bound to a protein complex composed of COX20, COA6, SCO1, and SCO2. Copper is required to be transferred from COX17 to SCO1 where the oxidoreductase activity of SCO2 on COX2 cysteine is considered fundamental. Likewise, these reactions are associated with COA6 which reduces COX2 and SCO1, allowing copper binding (Cobine et al., 2021). The network suggests that the interaction of COA6 with COX17, SURF1, SCO1, SCO2, and MIA4 (CHCHD4) appears to be the entry point for cysteine-containing chaperones such as COA6, COX17, and COX19.

## Role of Copper on Mitochondria and Cell Fate Determination

Hematopoietic stem cells (HSCs) and their fate are highly dependent on the mitochondrial metabolism. Mitochondria are responsible for metabolic reprograming needed for the commitment and differentiation of HSCs into different cell lineages, beyond energy production. In erythropoiesis, mitochondria are also involved in heme and hemoglobin biosyntheses (Fontenay et al., 2006; Kim et al., 2008; Chung et al., 2012). Typical mammalian HSCs remain quiescent in the hypoxic zone of the bone marrow (endosteal niche) to favor self-renewal, a process governed by the stabilization of the hypoxia-inducible factor-1 alpha (HIF-1α) protein and consequent heterodimerization with HIF-1ß to activate transcription in many target genes, including glycolytic enzymes (Takubo et al., 2010). Thus, HSCs and embryonic stem cells have a minimal basal metabolism relying mainly on glycolysis, while mitochondrial respiration is low to avoid the generation of reactive oxygen species (ROS) (Zhang et al., 2011; Xu X. et al, 2013; Bigarella et al., 2014). Transition from glycolysis to OXPHOS and then from self-renewal to differentiation is dependent on the presence of oxygen (Piccoli et al., 2005; Piccoli et al. 2006; Piccoli et al. 2013), mitochondrial respiratory substrates (Zhang et al., 2011; Vozza et al., 2014), and the generation of mitochondrial ROS (Maryanovich and Gross 2013; Bigarella et al., 2014). Furthermore, the participation of mitochondrial fusion and fission events, the so-called mitochondrial dynamics (MtDy) (Wanet et al., 2015; Luchsinger et al., 2016; Gonzalez-Ibanez et al., 2020), and the mitochondrial permeability transition pore (mPTP) (Folmes et al., 2012) have been involved in this metabolic reprogramming.

Since respiratory complex IV has two copper sites that are essential for its function, its activity can be easily manipulated by either copper removal/chelation or copper overload, affecting the overall purpose and assembly of the ETC. Thus, managing the cellular copper level seems a simple way for metabolic reprograming switching from glycolysis to OXPHOS, and *vice versa*, to control cell fate. In this regard, copper imbalances cause anemia, neutropenia, thrombocytopenia (decreased blood platelets), myelodysplastic syndrome, and leukemia due to alterations in the differentiation process of the hematopoietic progenitor cells (Zidar et al., 1977; Goyens et al., 1985; Hirase et al., 1992; Gregg et al., 2002; Fong et al., 2007; Halfdanarson et al., 2008; Griffith et al., 2009; Gletsu-Miller et al., 2011; Gabreyes et al., 2013). In hematopoietic stem cells (HSCs), low copper delayed differentiation and stimulated cell expansion. Since the lack of copper promotes cell expansion, it has been proposed to be useful for *ex vivo* stem cell expansion which is yearned for cell therapy (Peled et al., 2002; Huang et al., 2009; Dahlberg et al., 2011). At the mitochondrial level, the use of copper chelators results in decreased COX levels and mitochondrial and cellular ROS levels, the activation of glycolysis, and the appearance of giant mitochondria in erythropoietic cell lines and primary EPO-induced CD34 ^+^ cell differentiation (Dallman and Goodman 1970; Wakabayashi et al., 1975; Kim et al., 2010; Bustos et al., 2013; Ruiz et al., 2014; Jensen et al., 2019). This metabolic change could allow their survival and cellular proliferation while awaiting the ideal conditions to continue their differentiation (Bustos et al., 2013; Jensen et al., 2019). Copper deficiency alters metabolic reprogramming in differentiating HSCs, reversing to an immature phenotype related with erythroid progenitor cell expansion, with low oxygen consumption, low ROS generation, and high membrane potential (Jensen et al., 2019). In addition, copper deficiency has been associated with increased expression of the mitochondrial fusion proteins such as MFN1, MFN2, and OPA1 (optic atrophy 1) (Yoon et al., 2003; Chang and Blackstone 2010), and increased mitochondrial membrane potential in K562 and EPO-induced CD34 ^+^ cells (Bustos et al., 2013; Jensen et al., 2019). In our laboratory, we generated a model of copper deficiency in C57 black mice with a BCS treatment that showed mild signs of anemia (Ruiz et al., 2014) and reduced the expression of ceruloplasmin, ATP7B, and CIV, as previously reported by other groups (Liebes and Medeiros 1997; Medeiros et al., 1997; Rossi et al., 1998; Cerone et al., 2000; Getz et al., 2011; Gupta et al., 2011; Lassi and Prohaska 2012; Ruiz et al., 2014). Also, the BCS-treated mice had more oxidized proteins, upregulation of the MFN2 protein, OXPHOS remodeling from supercomplexes to individual complexes, and higher oxygen consumption rates (Ruiz et al., 2014). Besides, the liver showed large mitochondria with a mix of average, balloon, and butternut squash mitochondria (Ruiz et al., 2014). The butternut squash mitochondria showed features between normal and swollen mitochondria, which are likely an intermediate state between the two phenotypes (Ruiz et al., 2014). It has also been reported that copper depletion with TEPA (tetraethylenepentamine) favored the maintenance and expansion of HSCs for erythroid differentiation (Huang et al., 2009).

On the other hand, individuals with high levels of copper in their blood showed altered hematological parameters with an increased number of proerythroblasts (early stage of erythropoietic differentiation) and a decreased number of orthochromatophilic erythroblasts (advanced phase of erythropoietic differentiation) (Gregory and Eaves 1978). High copper content has been associated with hemolysis because of its direct effect on the ETC and increased generation of oxidative stress (Fibach and Rachmilewitz 2008; Ruiz et al., 2016). This could trigger a compensatory mechanism by accelerating the erythropoietic process and releasing immature cells into the bloodstream (Wagner et al., 2000). High copper levels are then associated with mitochondrial dysfunction and cell death. However, noncytotoxic copper overload has been described to improve mitochondrial function by stimulating the biogenesis and assembly of complex IV along with the generation of a physiological amount of ROS in the erythropoietic cell line K562. This process stimulated mitochondrial turnover by mitophagy and mitochondrial biogenesis, resulting in highly active mitochondria overpacked with OXPHOS proteins for ATP synthesis (Ruiz et al., 2016). Thus, physiological ROS generation has been considered an adaptive response to produce adjustments in signaling pathways related to proliferation, metabolic adaptation, cell motility, adaptations to hypoxia, and angiogenesis (Wang et al., 2018).

**FIGURE 4:**
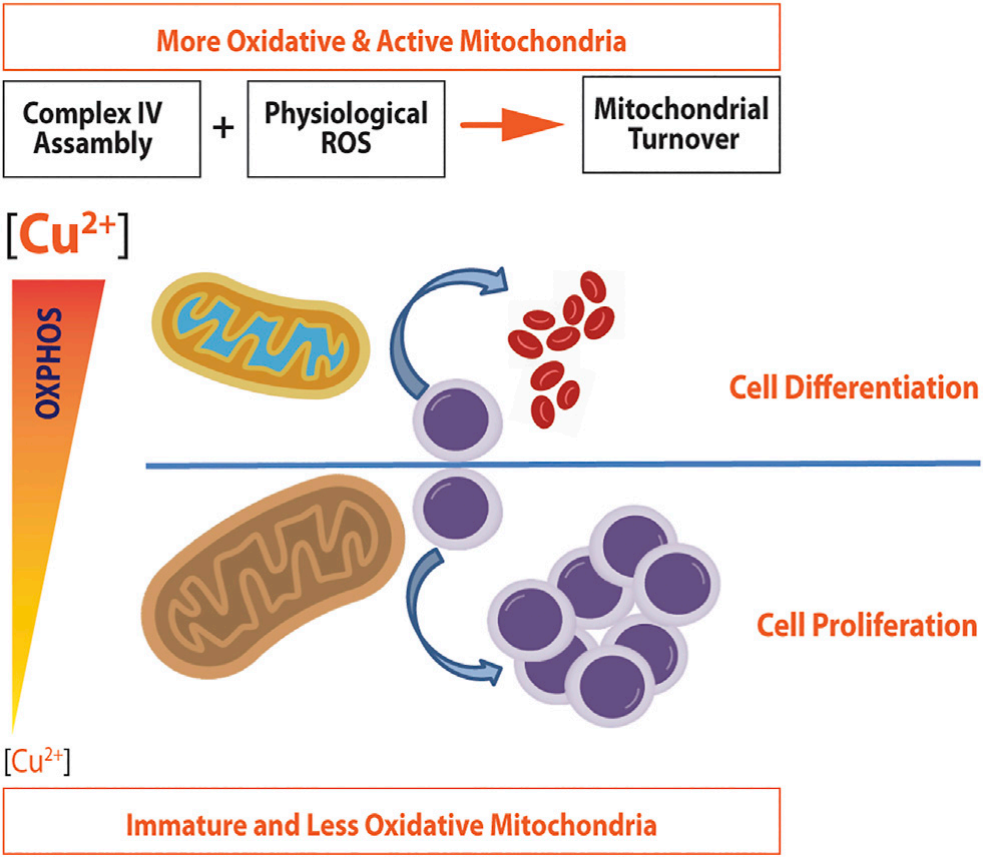
Metabolic reprogramming induced by copper in hematopoietic stem cells. Noncytotoxic copper overload induces both the complex IV assembly and ROS generation, which promote mitochondrial turnover and biogenesis. As a result, more active and oxidative mitochondria are produced, which promote cell differentiation in HSCs. A decrease in copper concentration will down regulate the expression of complex IV, making mitochondria less oxidative. Since mitochondria will not be able to satisfy energy demands, the energy metabolism switches to glycolysis, which in turn will promote cell proliferation (adapted from Jensen et al., 2019 and Ruiz et al., 2016).

Cellular copper levels, either in excess or lack, altered mitochondrial function in stem cells and many other cell types having an impact on either proliferation or differentiation. Figure 4 represents the metabolic reprogramming of HSCs toward expansion or differentiation according to intracellular copper levels. A lack of copper will induce cell expansion, a noncytotoxic copper overload, and differentiation. Furthermore, Tables 2, 3 show several primary cells and cell lines that have been exposed to different copper concentration or chelators and assayed for cell proliferation or differentiation.

**TABLE 2:** Effect of copper increase on proliferation and differentiation in different cell types.

Cells	[Cu]	Features	Proliferation	Differentiation	References
**Studies in primary cells**					
MSC (mesenchymal stem cells)	50 µM Cu-His	Present in the mesodermal germinal layer	↓	↑ into osteoblasts or adipocytes	Rodríguez et al. (2002)
MEFs (Mouse embryonic fibroblasts)	10 µM CuCl_2_	Synthesize fibers and maintain matrix	↑	−	Itoh et al. (2008)
HUVEC	500 µM CuSO_4_	Human umbilical vein endothelial cells	↑	−	Hu (1998)
HPC (CD34^+^)	10 µM CuCl_2_	Hematopoietic progenitor cells	↓	↑	Peled et al. (2002); Peled et al. (2005)
HSC	10 µM CuCl_2_	Hematopoietic stem cells	↓	↑	Huang et al. (2009)
RIP1–Tag2 mice	20 µM Cu *via* drinking water from 4 to 15 weeks of age	Tumorigenetic mouse model of pancreatic islet cell carcinoma	↑ promotes tumor growth	−	Ishida et al. (2013)
Astrocytes	30 μM CuCl_2_	Astroglia from rat cerebral cortex	↑	−	Hu et al. (2016)
BMECs	30–120 µM CuCl_2_	Brain microvascular endothelial cells from rat	↑	−	Wang et al. (2016)
**Studies in cell lines from cancer**					
HepG2	64 µM CuSO_4_	Human liver hepatocellular cells	↓	−	Aston et al. (2000); Arnal et al. (2012)
K562	95 µM CuCl_2_	Human erythroleukemia cell line	↑	↓	Ruiz et al. (2016)
A-549	80 µM Cu	Adenocarcinomic human alveolar basal epithelial cells	↓	−	Arnal et al. (2012)
HL-60	12 μM Cu(NO_3_)_2_	Human promyelocytic leukemia cells	↓	↑	Bae and Percival (1993)
Huh-7 and OUMS29	300 µM CuSO_4_	Human hepatoma cell lines	↓	−	Oe et al. (2016)
A-549	5–15 µM CuCl_2_	Adenocarcinomic human alveolar basal epithelial cells	↑	−	Zhang et al. (2016)

↑: increase; ↓: decrease.

**TABLE 3:** Effect of copper decline on proliferation and differentiation in different cell types.

Cells	[Chelator]	Features	Proliferation	Differentiation		References
**Studies in primary cells**						
Human fibroblast	100 µM d-penicillamine and 8 µM CuSO_4_	Synthesize fibers and maintain the extracellular matrix of tissues	↓		−	Matsubara and Hirohata (1988); Goodman et al. (2004)
HSC	40 µM Tepa	Hematopoietic stem cells	↑		↓	Huang et al. (2009)
HPC	40 µM Tepa	Hematopoietic progenitor cells	↑		↓	Peled et al. (2002); Peled et al. (2005); Huang et al. (2009)
CD34 ^+^ hematopoietic stem cells	10, 20, or 30 µM BCS during 6 days of culture 1 × 10^−4^% or 5 × 10^−4^% sodium azide during 6 days of culture	HSC possesses multipotentiality, enabling them to self-renew and to produce mature blood cells, such as erythrocytes, leukocytes, platelets, and lymphocytes	↑		↓	Jensen et al. (2019)
Lymphocyte T	25 μg/ml d- penicillamine and 2 μg/ml CuSO_4_	Immune system cell	↓		−	Lipsky and Ziff (1980); Lipsky (1984)
HUVEC	Inhibition of Cu transporter Ctr1	Human umbilical vein endothelial cells	↓		↑	Narayanan et al. (2013)
RIP1–Tag2 mice	Tetrathiomolybdate 1 mg daily for up to 3 weeks	Tumorigenetic mouse model of pancreatic islet cell carcinoma	↓		↓ reduction in the number of angiogenic islets	Ishida et al. (2013)
βTC3	10 µM tetrathiomolybdate	Derived from RIP1–Tag2 tumors	↓		−	Ishida et al. (2013)
Tumor endothelia	Penicillamine (2000 µg per dose per mouse) Trientine (700 µg per dose per mouse)	Derived from mesothelioma tumors in mice	↓		−	Crowe et al. (2013)
A549 xenograft	Curcumin (U0126)	Tumor xenograft model	↓		−	Zhang et al. (2016)
**Studies in cell lines from cancer**						
K562	10 µM BCS	Human erythroleukemia cell line	↓		↓	Bustos et al. (2013)
9L Gliosarcoma rat model	2 mg d- penicillamine orally, once daily, on the 3 days before and after implantation	Brain Glial cell	↓		−	Yoshida et al. (1995)
U937	5 μmol/L tet (2,3,2-tetraamine)	Human promonocytic cells	↓		↓	Huang and Failla (2000)
A2780	10 µM tetrathiomolybdate	Human ovarian carcinoma cells	↓		−	Ishida et al. (2013)
A375	Sulfur nanoparticles (Nano-S)	Malignant melanoma	↓		−	Liu et al. (2016)
MCF-7	Nano-S	Breast cancer cells	↓		−	Liu et al. (2016)
H1299	DC_AC2, DC_AC30, DC_AC49, DC_AC50, DC_AC61, DC_AC71	Lung cancer cells	↓		−	Wang et al. (2015)
212LN	DC_AC2, DC_AC30, DC_AC49, DC_AC50, DC_AC61, DC_AC71	Head and neck cancer cells	↓		−	Wang et al. (2015)
MB231	DC_AC2, DC_AC30, DC_AC49, DC_AC50, DC_AC61, DC_AC71	Breast cancer cells	↓		−	Wang et al. (2015)

↑: increase; ↓: decrease.

## Copper Role in Cancer

Metabolic reprogramming can go from differentiated to proliferative cells as it happens in cancer cells and induced pluripotent stem cells (iPSCs), where the cell metabolism switches from OXPHOS to aerobic glycolysis (Warburg effect). Regarding mitochondria in cancer cells, they displayed a stem cell-like phenotype, that is, low oxygen consumption and low ROS, although they displayed higher membrane potential than healthy cells (Bonnet et al., 2007; Hanahan and Weinberg Robert 2011; Schulze and Harris 2012; Wanet et al., 2015).

The remarkable change in the metabolism that occurs in cancer cells has been associated, among other factors, with the management of copper and proteins that use it (Gupte and Mumper 2009). An example of this is obtained by observing the high levels of ceruloplasmin found in various forms of cancer, such as lymphomas, breast cancer, and gastrointestinal cancer (Scanni et al., 1977; Ungar-Waron et al., 1978; Linder and Roboz 1986; Gupte and Mumper 2009), and ceruloplasmin levels have been found to be increased 4 to 8 times during malignant progression, returning to normal levels after tumor regression (Brem et al., 1990; Brem 1999). Copper levels increase three times in blood plasma, relating to tumor burden, progression, incidence, invasion, and reoccurrence of the disease (Goodman et al., 2004; Gupte and Mumper 2009). The human Cu proteome, with 54 Cu-binding proteins, are up- or downregulated in 18 cancer types (thymus, head and neck, esophagus, adrenal gland, bladder, stomach, soft tissue, kidney, pancreas, bile duct, liver, prostate, cervix, breast, uterus, thyroid, colorectal, and lung), showing intricate patterns (Blockhuys et al., 2017).

Angiogenesis is the development of new blood vessels which are required for tumor growth (Folkman and Klagsbrun 1987). Cancer cells have developed ways to synthesize and release their own angiogenic stimulants or to recruit endothelial cells for the same purpose (Goodman et al., 2004; Lowndes and Harris 2004; Lowndes and Harris 2005; Gupte and Mumper 2009).

Copper is a potent stimulator of the angiogenic process by the activation of angiogenic factors like the interleukin (IL)-1, IL-6, and IL-8; tumor necrosis factor alpha (TNF-α); angiogenin; the basic fibroblast growth factor (bFGF); fibronectin; and the vascular endothelial growth factor (VEGF), which are critical for tumor angiogenic developments (Li 2020). Copper sulfate (CuSO_4_) combined with increasing doses of VEGF or FGF-2 revealed synergistic effect in endothelial cells grown in a 3D culture system with enhanced collagen fiber deposition providing complexity of angiogenic networks (Gérard et al., 2010). Copper-coated disks inserted subcutaneously in rats stimulated a high and extended-release of interleukin-1α (IL-1α) and low quantities of IL-1β by recruited cells (Suska et al., 2003). HIF-1 transcriptional activity requires the accumulation of HIF-1α following inhibition of prolyl hydroxylases. This inhibition is generated by hypoxia, and chemical hypoxia can be generated by cobalt. Copper chelation with tetraethylenepentamine alters the transcriptional activity of HIF-1α, decreasing the mRNA and protein expression of VEGF, which is rescued by 25 μM CuSO4. Also, CCS1 gene silencing blocked VEGF expression, revealing copper requirement for cobalt-activated transcriptional activity of HIF-1α in promoting the expression of VEGF (Qiu et al., 2012). Angiogenin-bound copper is a strong inducer of blood-vessel development, and it binds to endothelial cell receptors and extracellular matrix components (Soncin et al., 1997). *In vitro*, copper sulfate incubation stimulates human umbilical vein endothelial cell proliferation and migration (Hu 1998). *In vitro*, copper salts could induce the synthesis of the fibronectin matrix associated with angiogenesis (Kochi et al., 1983; Gullino 1986). The study of the mechanism of angiogenesis in the adult organism, through a rabbit cornea model, implanted with 10–75°μg of copper sulfate pellets in the corneal stroma, induced long-lasting and dose-dependent neovascularization, showed that copper directly stimulates angiogenesis, and it is helpful in the evaluation of antiangiogenic agents (Parke et al., 1988). The expression of some angiogenic growth factors and cytokines is reduced when copper levels are decreased (Brem et al., 1990; Brem 1999). Cerebral neoplasms sequester copper, utilizing a rabbit brain tumor model, and showed that normocupremic animals formed big vascularized VX2 carcinomas, while in copper depleted by diet- and penicillamine (CDPT)-treated rabbits, the tumors developed were small, circumscribed, and relatively avascular. Metabolic and pharmacologic removal of copper overcomes cerebral tumor angiogenesis (Brem et al., 1990).

With these antecedents, the concept of antiangiogenic therapies as cancer treatments has begun, although a phase two trial of copper depletion by diet and penicillamine as antiangiogenic therapy of glioblastoma multiforme did not improve survival in patients (Brem 1999; Brem et al., 2005). Since it was discovered that tumor development is dependent on the formation of new blood vessels, the mechanisms that regulate angiogenesis continue to be investigated (Tisato et al., 2010).

Many angiogenic promoters appear to be dependent on copper concentrations. They control various endogenous stimulators by acting as a cofactor, leading to the use of copper chelators as therapeutic strategies with antiangiogenic function. The most used copper chelators in tests are D-Pen (d-penicillamine) and TM (tetrathiomolybdate). D-Pen is an effective copper chelator and has antiangiogenic properties. In addition, it can inhibit essential growth factors (VEGF and FGF), requiring copper as a cofactor (Brem et al., 1990; Brem 1999; Gupte and Mumper 2009). However, in the phase two trial, two-month treatment with a copper-deficient diet combined with D-pen as antiangiogenic therapy for glioblastoma did not significantly increase the patients’ survival, despite effective hypocupremia (Brem et al., 2005).

On the other hand, trials in rodents showed that by decreasing copper levels using TM, there was a sharp decrease in angiogenesis (Brewer 2001). A phase two trial with TM in patients with advanced kidney cancer concluded that this treatment was well tolerated, decreased copper concentrations in all patients, and that 31% of patients showed stability in the disease for at least 6 months (Redman et al., 2003). TM reduces the secretion of IL-6 and bFGF by head and neck SCC (HNSCC) cell lines *in vitro*. Also, in a rat aortic ring assay, the HNSCC cell lines treated with TM decreased endothelial cell chemotaxis, tubule formation, and neovascularization (Teknos et al., 2005). TM induced mild copper deficiency in the phase I clinical trial in patients with metastatic cancer treated with a dose of 120 mg/day, followed by ceruloplasmin decrease to 20% baseline without toxicity (Brewer et al., 2000). Then, a phase II clinical trial in patients with malignant pleural mesothelioma received TM at 180 mg/day, starting 4–6 weeks post-surgery for 15 months. After 34 days, this dosage decreased ceruloplasmin levels from 45 mg/dl to 13 mg/dl. This mild copper deficiency decreases VEGF levels in the serum from an average of 2086 to 1,250 pg/ml, and patients showed encouraging results about overall survival (Pass et al., 2008). There must be several mechanisms by which the decrease of copper using TM inhibits tumor angiogenesis. For example, TM-treated SUM149 (inflammatory breast cancer cell line) cells released significantly lower amounts of 5 angiogenic factors (VEGF, FGF, IL1, IL6, and IL8) than untreated cells. TM has also shown to inhibit endothelial cell differentiation and to suppress NFκB protein levels and its transcription (NFκB is known to regulate many genes involved in tumor invasion, angiogenesis, and metastasis) (Pan et al., 2002; Brewer 2003; Brewer et al., 2003).

In the murine HCC (human hepatocellular carcinoma) xenograft model, tumor development and angiogenesis were suppressed by trientine, a copper-chelating agent. Trientine treatment in combination with a copper-deficient diet caused a noticeable inhibition of neovascularization and increased apoptosis in the HCC tumor (Yoshii et al., 2001).

The depletion of mitochondrial copper induces a metabolic reprogramming that shifts from oxidative to glycolytic metabolism and reduces energy production. The above is an effective therapy against cancer types that depends on OXPHOS. Copper-depleting nanoparticle (CDN) targeted on mitochondria causes a metabolic shift from respiration to glycolysis in triple-negative breast cancer (TNBC). CDNs are composed by a copper-depleting moiety (CDM) and a semiconducting polymer nanoparticle (SPN). The CDM is composed of N,N-Bis (2-pyridinylmethyl)-1,2-ethanediamine linked to tricarbocyanine. SPN consists of semiconducting polymers and phospholipid-polyethylene glycol (PEG). CDN administration inhibits tumor growth and improves survival of three mouse models of TNBC (Cui et al., 2021).

Abnormal copper accumulation in cancer cells can help to distinguish transformed cells from healthy ones and can be used as targets for novel chemotherapeutic agents (Daniel et al., 2004), such as the use of organic copper compounds in antitumor treatments has been investigated as cytotoxic agents and showed antitumor activity, such as Cu(II) thiosemicarbazide complexes, Isatin–Schiff base Cu(II) complexes [(4,7-dimethyl-1,10-phenanthroline) (glicinate)], Cu(II) nitrate complex, Casiopeina II-gly, and imidazole, benzimidazole, and pyrazole Cu(II) complexes. The mode of action of these compounds is through producing high levels of ROS, mitochondrial toxicity, and DNA interactions, inhibiting cell proliferation and producing apoptosis. For example, Casiopeínas® are mixed chelate copper (II) compounds ([Cu(N-N) (O-O)]NO3 or [Cu(N-N) (O-N)]NO3) with antitumor potential. Casiopeínas® alter mitochondria bioenergetics, with reports of inhibition of respiration and ATP synthesis, mitochondrial swelling, loss of mitochondrial membrane potential, and cytochrome c release (Ruiz-Azuara and Bravo-Gomez 2010). Another example of this is found in the use of D-Pen together with cupric sulfate. This mixture was able to cause a dose-dependent cytotoxicity in human cancer cells. (Gupte and Mumper 2009).

Cancer cells are sensitive to proteasome inhibition, suggesting good potential as anticancer agents for copper complexes (Zuo et al., 2013). Amino acid Schiff base–copper (II) complexes inhibit the chymotrypsin-like activity of the proteasome, producing a buildup of proteasome target proteins Bax and IκB-α, therefore inducing growth inhibition and apoptosis. Cyclic dithiocarbamate (DTC) ligands, such as the neutral Cu(II) derivatives of the type [Cu(DTC)2], show remarkable anticancer activity (IC50≤1 µM) (Brustolin et al., 2018). Cu(II) ion increases the activity of 8-hydroxyquinoline derivatives in inhibiting the chymotrypsin-like activity of the proteasome and induces growth inhibition and apoptosis (Oliveri et al., 2017). The Cu(II) ion complex with glycoconjugate DTC shows potential applications in targeted chemotherapy; in particular, the CuGlu ([CuII(DTC-β-d-glucose)2]) revealed an exciting IC50 (2.0 ± 0.1 µM) value for the HCT116 human colorectal carcinoma cell line (Pettenuzzo et al., 2019). Estrogen-functionalized Cu(II) complexes are potent anticancer agents with low IC50 values. Their cellular uptake occurred *via* passive diffusion, and their mechanisms involved high DNA intercalation, intense DNA cleaving activity, and stimulation ROS production (Barrett et al., 2020). [Cu(l-proline methyl ester DTC)2] copper compounds into micelles with a cancer-targeting biomolecule are presented as a proper “Trojan Horse” strategy for the delivery of Cu-DTC chemotherapeutics (Brustolin et al., 2020).

## Mechanisms of Copper Action on Tumor Development

It has been shown that exposure to high levels of copper in water (1.3 mg/L maximum levels allowed in public water supplies) (Council et al., 2000) can enhance tumor progression but not of forming new tumors and decreasing the intake of this metal stops the development of the disease, indicating that copper is not capable of producing transformation but is a necessary nutrient for these cells. That is why copper has been described as a stimulator of tumor development and not an initiator of transformation, as it promotes several processes related to cancer (Ishida et al., 2013).

Several types of cancer cells possess very high levels of the transmembrane transporter CTR1, explaining how these cells obtain the high concentrations of copper. Cancer cells treated with copper chelator treatments have dramatically decreased ATP production even when there is an increase in glycolysis, indicating that these cells still rely on OXPHOS. Thus, these elevated levels of copper can regulate the production of ATP by OXPHOS to meet the demands of the accelerated proliferation found in solid cancers, indicating that copper may be a limiting nutrient for tumor development (Ishida et al., 2013). The high copper content could be activating the function as a transcription factor of ATOX1, further enhancing cell proliferation (Itoh et al., 2008).

ATOX1 is established to perform a vital function in copper homeostasis (Hatori and Lutsenko 2016). Moreover, ATOX1 shows high expression in different cancers (Cai and Peng 2013; Blockhuys et al., 2017) and has been described to present a critical function in angiogenesis (Chen et al., 2015). Remarkably, ATOX1 is a copper-dependent transcription factor (requires copper binding) that stimulates the expression of NADPH oxidase p47phox and Cyclin D1, relating to increased ROS generation and proliferation, respectively (Hamza et al., 2001; Itoh et al., 2008; Chen et al., 2015). The nuclear localization of ATOX1 is associated with the severity of metastatic colorectal cancer. Stimulation of colon cancer metastasis with activin A promotes ATOX1 nuclear translocation in metastatic SW620 and nonmetastatic SW480 colon cancer cell lines. Knockdown of ATOX1 in SW620 decreased ROS generation and colony formation through a decreased expression of NADPH oxidase p47phox and Cyclin D1 (Jana et al., 2020). Individual cell migration is an early step in breast cancer metastasis; thus, ATOX1 silencing decreases the breast cancer cell migration velocity *via* coordinated copper transport in the ATP7A-LOX (proenzyme of lysyl oxidase) axis (Blockhuys et al., 2020).

Considering possible copper effects on tumor development, the gene TP53—which encodes for the tumor suppressor protein p53—should be mentioned, as it is the most frequently mutated protein in human cancers (Kastenhuber and Lowe 2017). The protein p53 is a transcription factor that possesses a single zinc ion near its DNA-binding interface, and deficient zinc through competition with copper causes p53 to misfold, which results in functional loss of transcriptional activity (Loh 2010; Formigari et al., 2013).

Other mechanistic insight about copper action in cancer would be the oncogenic BRAFV600E that phosphorylates and activates the MEK1 and MEK2 kinases, which in turn phosphorylates and activates ERK1 and ERK2 kinases, stimulating the MAPK (mitogen-activated protein kinase) pathway to promote cancer. Alterations of the MEK1 interaction with copper and decrease of copper influx shooting down CTR1 decrease BRAFV600E driven signaling and tumorigenesis (Brady et al., 2014).

Moreover, the autophagy signaling is implicated in cellular proliferation, and copper is necessary for the activity of the ULK1 and ULK2 (ULK1/2) autophagic kinases, by directly binding Cu to ULK1/2. Elevated intracellular copper levels are related to starvation-induced autophagy and are enough to upregulate the ULK1 kinase activity and autophagic flux (Tsang et al., 2020). Copper-treated K562 cells showed reduced levels of protein P62, indicating an increased autophagic flux and mitochondrial fusion, which is restored to basal levels by TBAP (antioxidant molecule that mimics SOD2) treatment, suggesting that copper-induced ROS speeds up mitochondrial turnover (Ruiz et al., 2016). The loss of the copper transporter CTR1 decreased the growth and survival of KRASG12D-driven lung tumors, which are reliant on Cu-ULK1 binding and are coupled to reduced autophagy and signaling. This work established the molecular basis of copper chelation to prevent autophagy signaling, copper-dependent ROS generation (Fenton reaction), and glycolytic metabolism from limiting proliferation and improving cancer patients' survival (Tsang et al., 2020).

Interestingly, copper also has a role in regulating the programmed death ligand 1 (PD-L1), which is overexpressed by cancer cells to protect themselves from antitumor immune responses. Copper supplementation in cancer cells enhanced PD-L1 expression, driving cancer immune evasion, while copper chelation promoted ubiquitin-mediated degradation of PD-L1 and increased the number of tumor-infiltrating CD8+T and natural-killer cells (Voli et al., 2020).

**FIGURE 5:**
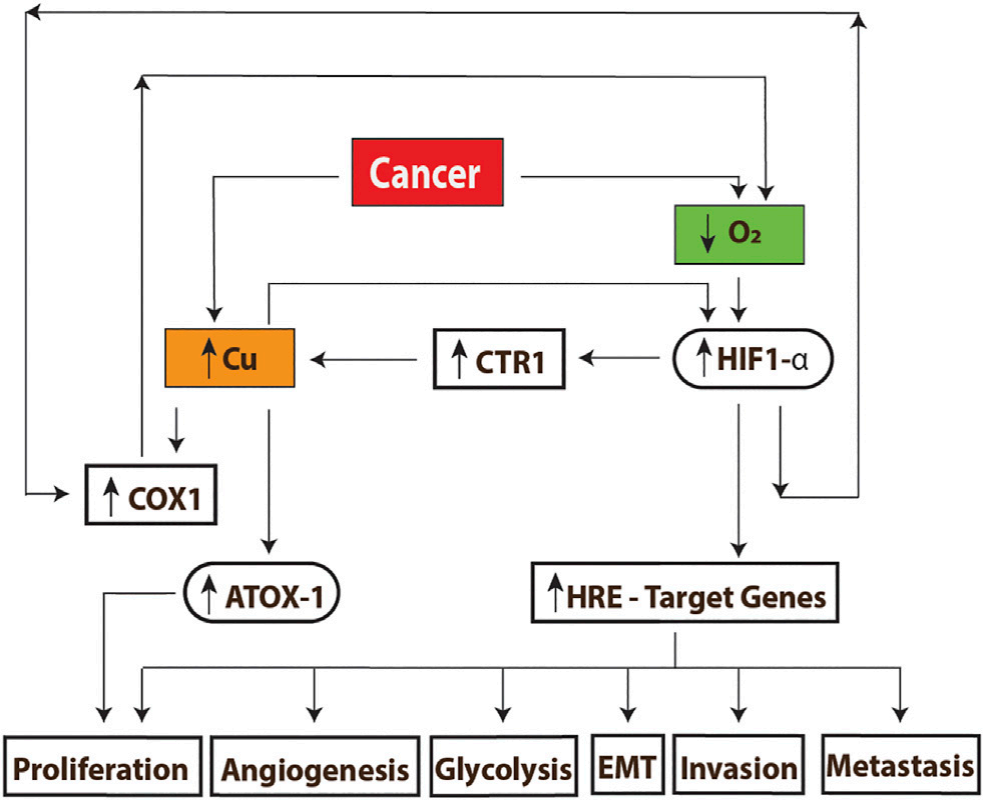
Mechanisms of copper action in cancer. **(A)** The high energy demand found in these cells decreases the oxygen concentrations, producing hypoxia; this causes an increase in the transmembrane transporter CTR1, which causes an increase of the intracellular concentrations of copper. The levels of this metal together with the reduced concentration of oxygen can stabilize and activate the HIF-1 transcription factor, triggering the activation of genes related to growth and tumor development. On the other hand, the high copper content promotes the assembly of complex IV (COX1) which is followed by the upregulation of OXPHOS. A higher oxygen demand will induce the upregulation of HIF1-alpha, creating a virtuous cycle for tumor growth. Excess of copper will also up regulate ATOX1 expression to promote cell proliferation as well. HRE: hypoxia-response Element. EMT: epithelial–mesenchymal Transition.

The presence of significant high serum copper levels in nonalcoholic fatty liver disease (NAFLD) in cirrhotic patients increased cell growth, migration, and invasion of liver cancer cells through the modulation of the MYC/CTR1 axis (MYC proto-oncogene, bHLH transcription factor/copper transport protein) (Porcu et al., 2018). Figure 5 summarizes the main findings of copper action in cancer.

## Final Thoughts

As largely discussed in this review, cellular copper concentration has a direct impact on cell proliferation, differentiation, or cell death. Copper property to control cell fate has been exploited by modern medicine in the field of stem cell–based regenerative medicine as well as in the fight against cancer. The main action of copper is at the level of mitochondrial function and metabolism because copper is a prosthetic group of respiratory complex IV. Copper bioavailability has been shown to regulate both expression and activity of complex IV and then the balance between glycolysis and OXPHOS. Copper deficiency will favor glycolysis and cell proliferation, and a noncytotoxic copper overload, OXPHOS, and cell differentiation. However, the final outcome will be tissue- or cell-specific. Changes in mitochondrial metabolism also impact the amount intermediate metabolites, such as acetyl-CoA, citrate, α-ketoglutarate, ROS, and NAD+/NADH among others, which have a direct effect on the transcriptional and epigenetic regulation of cells, by means of mitochondria–nucleus retrograde communication. Copper-induced transcriptional regulation also occurs *via* ATOX1, a copper-dependent transcription factor associated with angiogenesis and tumor development. On the other hand, an excessive copper overload will induce a massive amount of ROS, due to Fenton and Haber–Weiss reactions, causing cell death *via* apoptosis or necrosis. Thus, copper action involves either going from mitochondrial-induced metabolic reprogramming to genetic reprogramming to change cellular phenotype, or cell death.

Pharmacological modulation of intracellular copper concentrations is effective to control stem cell expansion and/or differentiation for regenerative medicine as well as to control tumor growth and cancer development. Cancer cells have been shown to accumulate more copper than healthy cells and to be sensitive to proteasome inhibition. Therefore, many copper-based pharmacological strategies have been developed to eliminate cancer cells. Those include copper chelators, copper-depleting nanoparticles, and copper-based compounds which have been shown to be successful both *in vitro* and *in vivo*. Nevertheless, more basic and clinical research is still needed regarding cell-specific targeting and delivery of those copper compounds.

The study of copper on mitochondrial function and metabolism has opened a powerful and novel route for drug discovery and nanobiotechnology, not only for treating catastrophic diseases like cancer but also for protecting and/or improving mitochondrial activity and capacity. Remarkably, proper mitochondrial function is associated with healthy aging and the delay of aging-associated diseases.

## Abbreviations

ATOX1, antioxidant 1 copper chaperone; CDM, copper-depleting moiety; CDN, copper-depleting nanoparticle; COX, cytochrome C oxidase, complex IV; CTR1, copper transport protein; CuL, low molecular weight copper ligand; D-Pen, d-penicillamine; ETC, electron transport chain; HCC, human hepatocellular carcinoma; HSCs, hematopoietic stem cells; IMS, mitochondrial intermembrane space; MYC, MYC proto-oncogene; BHLH transcription factor; OXPHOS, oxidative phosphorylation system; PMF, proton motive force; SOD1, copper/zinc superoxide dismutase; SOD2, Mn-superoxide dismutase; SPN, semiconducting polymer nanoparticle; TEPA, tetraethylenepentamine; TM, tetrathiomolybdate.
