# Supplementary material for: Role of Copper on Mitochondrial Function and Metabolism
Source: Front Mol Biosci. 2021 Aug 24;8:711227. doi: 10.3389/fmolb.2021.711227 (PMC8421569; doi:10.3389/fmolb.2021.711227)
Supplement: Supplementary file 1 [file DataSheet1.docx]

Supplementary Material

# Supplementary Data Figure 3

Figure 3 Legend. Copper protein network in mitochondria- STRING interaction network. The interaction network was created with the STRING (Search Tool for the Retrieval of Interacting Genes/Proteins) database version 11.0. A medium confidence cutoff of 0,7 was implemented in this work. The resulting protein association network for copper was visualized by a Cytoscape v3.8.2. Proteins are presented as nodes connected by lines (Edge) whose thickness represent the strength of the connection based on the STRING database. Nodes (Shared named, Stringdb canonical name, Display name, Neighborhood connectivity -number of neighbors-). COX17 (9606.ENSP00000261070, Q14061, COX17, 7); COX11 (9606.ENSP00000299335, Q9Y6N1, COX11, 7); SURF1 (9606.ENSP00000361042, Q15526, SURF1, 8); PET191 (9606.ENSP00000330730, Q86WW8, COA5, 8); SCO1 (9606.ENSP00000255390, O75880, SCO1, 8); MT-CO2 (9606.ENSP00000354876, P00403, MT-CO2, 8); COA6 (9606.ENSP00000355572, Q5JTJ3, COA6, 8); MIA40 (9606.ENSP00000295767, Q8N4Q1, CHCHD4, 8); COX19 (9606.ENSP00000342015, Q49B96, COX19, 9); SCO2 (9606.ENSP00000444433, O43819, SCO2, 9); MT-CO1 (9606.ENSP00000354499, P00395, MT-CO1, 9); COX23 (9606.ENSP00000306425, Q9BUK0, CHCHD7, 8); COX20 (9606.ENSP00000406327, Q5RI15, COX20, 9); SOD1 (9606.ENSP00000270142, P00441, SOD1, 9); MT-CO3 (9606.ENSP00000354982, P00414, MT-CO3, 9); CCS (9606.ENSP00000436318, O14618, CCS, 6); PARK7 (9606.ENSP00000418770, Q99497, PARK7, 4); SLC25A3 (9606.ENSP00000228318, Q00325, SLC25A3, 0); SLC25A28 (9606.ENSP00000359526, Q96A46, SLC25A28, 0); SLC25A37 (9606.ENSP00000429200, Q9NYZ2, SLC25A37, 0).

The network clustering coefficient is the average of the clustering coefficients for all nodes in the network. Here, nodes with less than two neighbors are assumed to have a clustering coefficient of 0. Nodes (Clustering coefficient). COX17 (0,47); COX11 (0,45); SURF1 (0,61); COA5 (0,61); SCO1 (0,61); MT-CO2 (0,67); COA6 (0,64); CHCHD4 (0,61); COX19 (0,81); SCO2 (0,67); MT-CO1 (1,0); CHCHD7 (0,8); COX20 (0,67); SOD1 (1,0); MT-CO3 (0,70); CCS (0,33); PARK7 (0); SLC25A3 (0); SLC25A28 (0); SLC25A37 (0).

Closeness centrality is a measure of how fast information spreads from a given node to other reachable nodes in the network. The closeness centrality of a node measures its average farness (inverse distance) to all other nodes. Nodes with a high closeness score have the shortest distances to all other nodes. Nodes (Closeness centrality). COX17 (0,80); COX11 (0,76); SURF1 (0,67); COA5 (0,67); SCO1 (0,67); MT-CO2 (0,64); COA6 (0,64); CHCHD4 (0,64); COX19 (0,62); SCO2 (0,62); MT-CO1 (0,57); CHCHD7 (0,55); COX20 (0,53); SOD1 (0,52); MT-CO3 (0,52); CCS (0,52); PARK7 (0,35); SLC25A3 (0); SLC25A28 (0); SLC25A37 (0).

Edge confidence (Stringdb score): low (0.150); medium (0.400); high (0.700); highest (0.900). Stringdb score: COX17 (pp) COA6: 0,703; SOD1 (pp) CHCHD4: 0,713; COX11 (pp) CCS: 0,716; COA6 (pp) SURF1: 0,718; COA5 (pp) SCO2: 0,719; COX19 (pp) COA6: 0,72; COX11 (pp) MT-CO3: 0,732; COA6 (pp) COX20: 0,737; MT-CO3 (pp) SURF1: 0,742; SCO1 (pp) COA5: 0,746; SURF1 (pp) COX20: 0,749; MT-CO2 (pp) COX20: 0,761; SCO1 (pp) COX20: 0,769; COA6 (pp) SCO2: 0,771; MT-CO1 (pp) SCO2: 0,776; COX17 (pp) SURF1: 0,783; COX11 (pp) CHCHD7: 0,79; SCO1 (pp) COA6: 0,795; COX11 (pp) MT-CO1: 0,799; COA5 (pp) COX20: 0,799; COA5 (pp) COA6: 0,81; CHCHD4 (pp) COX11: 0,812; COX11 (pp) COA5: 0,827; MT-CO2 (pp) SURF1: 0,831; SCO1 (pp) MT-CO1: 0,837; COX17 (pp) CCS: 0,847; COX11 (pp) MT-CO2: 0,847; MT-CO1 (pp) SURF1: 0,851; CHCHD4 (pp) COA5: 0,854; COX17 (pp) SOD1: 0,86; CHCHD7 (pp) COA5: 0,86; COX17 (pp) CHCHD7: 0,863; COX17 (pp) COA5: 0,874; MT-CO2 (pp) SCO2: 0,0877; SCO1 (pp) MT-CO2: 0,889; CHCHD7 (pp) COX19: 0,893; SCO1 (pp) COX19: 0,894; COX11 (pp) SURF1: 0,897; SURF1 (pp) SCO2: 0,9; COX17 (pp) MT-CO2: 0,901; COX11 (pp) SCO2: 0,907; COA5 (pp) COX19: 0,914; COX17 (pp) SCO2: 0,917; SCO1 (pp) SURF1: 0,921; SOD1 (pp) PARK7: 0,936; COX11 (pp) COX19: 0,939; COX17 (pp) COX11: 0,904; CHCHD4 (pp) COA6: 0,956; SCO1 (pp) COX17: 0,959; SCO1 (pp) COX11: 0,959; CHCHD4 (pp) CHCHD7: 0,963; COX17 (pp) COX19: 0,967; SOD1 (pp) CCS: 0,989; COX17 (pp) CHCHD4: 0,995; CHCHD4 (pp) COX19: 0,995; MT-CO2 (pp) MT-CO3: 0,999; MT-CO1 (pp) MT-CO3: 0,999.

**Table SI. Copper content in foods**

|  | **Food** | Copper mg in 100 g | Daily Value (DV) (%) |
| --- | --- | --- | --- |
| **High**: 20 % DV or more | Beef liver, cooked (FDC ID: 168627) | 14.59 | 729 |
|  | Veal, variety meats and by-products, liver, raw (FDC ID: 172534) | 11.86 | 593 |
|  | Mushrooms, shiitake (FDC ID: 168436) | 5.17 | 258 |
|  | Oysters, steamed (FDC ID: 1099136) | 4.84 | 241 |
|  | Cashew nuts (FDC ID: 554301) | 2.50 | 125 |
|  | Soybeans, mature seeds (FDC ID: 174270) | 1.66 | 83 |
|  | Sesame seeds (FDC ID: 1100608) | 1.40 | 70 |
|  | Peanuts, raw (FDC ID: 172430) | 1.14 | 57 |
|  | Crab, hard shell, steamed (FDC ID: 1099116) | 0.81 | 40 |
|  | Cheese, goat (FDC ID: 172197) | 0.63 | 31 |
|  | Tempeh (FDC ID: 174272) | 0.56 | 28 |
|  | Veal, variety meats and by-products, kidneys, raw (FDC ID: 174356) | 0.49 | 24 |
|  | Crustaceans, shrimp, raw (FDC ID: 175179) | 0.39 | 20 |
|  | Veal, variety meats and by-products, heart, raw (FDC ID: 172529) | 0.34 | 17 |
| **Good:** 10-19% DV | Beverages, cocoa mix, powder (FDC ID: 174122) | 0.29 | 14 |
|  | Prune, dried (FDC ID: 168162) | 0.28 | 14 |
|  | Guavas, raw (FDC ID: 1102666) | 0.23 | 11 |
|  | Veal, variety meats and by-products, brain, raw (FDC ID: 174351) | 0.22 | 11 |
|  | Durian, raw or frozen (FDC ID: 168192) | 0.21 | 10 |
|  | Potato, boiled (FDC ID: 1102882) | 0.20 | 10 |
|  | Tofu (Nigari) (FDC ID: 174291) | 0.20 | 10 |
|  | Avocados, raw (FDC ID: 1102652) | 0.19 | 9.5 |
|  | Chard, raw (FDC ID: 1103086) | 0.18 | 8.9 |
|  | Blackberries, raw (FDC ID: 1102700) | 0.17 | 8.2 |
|  | Pomegranates, raw (FDC ID: 1102695) | 0.16 | 7.9 |
|  | Litchis, raw (FDC ID: 169086) | 0.15 | 7.4 |
|  | Carambola, starfruit, raw (FDC ID: 171715) | 0.14 | 6.8 |
|  | Kiwifruit, green, raw (FDC ID: 327046) | 0.13 | 6.7 |
|  | Grapes, red or green (European type, such as Thompson seedless), raw (FDC ID: 174683) | 0.12 | 6.3 |
|  | Olives, green (FDC ID: 1103679) | 0.12 | 6.0 |
|  | Beef, ground, 97% lean meat/ 3% fat, raw (FDC ID: 173111) | 0.08 | 4.0 |
|  | Meat loaf, NS as to type of meat (FDC ID: 1099415) | 0.07 | 3.6 |
|  | Beef, cured, corned beef, canned (FDC ID: 170602) | 0.06 | 3.2 |
|  | Kale, raw (FDC ID: 1103116) | 0.05 | 2.6 |
|  | Radishes, raw (FDC ID: 169276) | 0.05 | 2.5 |

Daily Value (DV)= 2mg. USDA National Nutrient Database for Standard Reference. FDC ID (Food Data Central Identification). https://fdc.nal.usda.gov/index.html. ([Fink & Mikesky 2017](#_ENREF_46)). Copper Percent DV is calculated using the following formula: ((APS*100)/2) = %DV, APS = Amount Per Serving; 2 = Copper DV; %DV = Percent Copper. Copper levels in drinking-water range ≤0.005 to >30 mg/L, the major source is plumbing corrosion ([Council et al 2000](#_ENREF_31), [WHO 2004](#_ENREF_182)).

**Table SII. Copper content in Human Fluids (**[**Linder 1991**](#_ENREF_111)**,** [**Linder & Roboz 1986**](#_ENREF_112)**)**

| **Fluid** | **Copper**  **mg/d** |
| --- | --- |
| Blood | 6,0 |
| Bile | 2,5 |
| Gastric | 1,0 |
| Pancreatic | 0,5-1,3 |
| Perspiration | 0,3 |
| Duodenal | 0,3 |
